# Advantages of stereolithographic 3D printing in the fabrication of the Affiblot device for dot-blot assays

**DOI:** 10.1007/s00604-024-06512-z

**Published:** 2024-07-02

**Authors:** Jakub Novotny, Zuzana Svobodova, Marie Ilicova, Dominika Hruskova, Jana Kostalova, Zuzana Bilkova, Frantisek Foret

**Affiliations:** 1 Institute of Analytical Chemistry of the CAS, v. v. i., Veveri 967/97, 60200 Brno, Czech Republic; 2 Department of Biological and Medical Sciences, Faculty of Pharmacy in Hradec Kralove, Charles University, Pardubice, Czech Republic; 3 Department of Economy and Management of Chemical and Foodstuff Industry, Faculty of Chemical Technology, University of Pardubice, Pardubice, Czech Republic; 4 Department of Biological and Biochemical Sciences, Faculty of Chemical Technology, University of Pardubice, Pardubice, Czech Republic

**Keywords:** 3D printing, Microfluidics, Prototyping, Dot-blot, Antibody

## Abstract

In stereolithographic (SLA) 3D printing, objects are constructed by exposing layers of photocurable resin to UV light. It is a highly user-friendly fabrication method that opens a possibility for technology sharing through CAD file online libraries. Here, we present a prototyping procedure of a microfluidics-enhanced dot-blot device (Affiblot) designed for simple and inexpensive screening of affinity molecule characteristics (antibodies, oligonucleotides, cell receptors, etc.). The incorporation of microfluidic features makes sample processing user-friendly, less time-consuming, and less laborious, all performed completely on-device, distinguishing it from other dot-blot devices. Initially, the Affiblot device was fabricated using CNC machining, which required significant investment in manual post-processing and resulted in low reproducibility. Utilization of SLA 3D printing reduced the amount of manual post-processing, which significantly streamlined the prototyping process. Moreover, it enabled the fabrication of previously impossible features, including internal fluidic channels. While 3D printing of sub-millimeter microchannels usually requires custom-built printers, we were able to fabricate microfluidic features on a readily available commercial printer. Open microchannels in the size range 200–300 μm could be fabricated with reliable repeatability and sealed with a replaceable foil. Economic aspects of device fabrication are also discussed.

**Graphical abstract:**

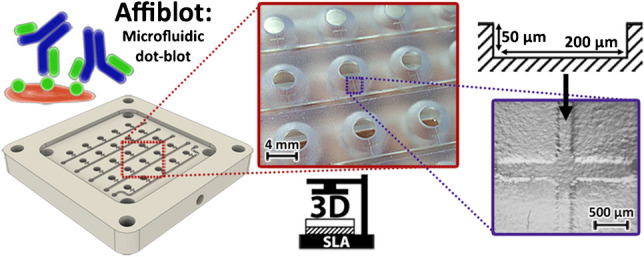

**Supplementary Information:**

The online version contains supplementary material available at 10.1007/s00604-024-06512-z.

## Introduction

Antibodies are used in a wide variety of fields of biological science, from immunohistochemistry to affinity chromatography. This generates high pressure on the reliability and reproducibility of antibodies. Antibody validation is a highly cited and discussed topic that suggests that a substantial portion of the antibodies available on the market are of poor quality [1–9].

According to our experiences, there are significant differences in the affinity/avidity and cross-reactivity of antibodies obtained from different sources as was reported in our previous paper [10]. Therefore, for fast affordable parallel screening of antibodies from various suppliers, a dot-blot-based device was designed and manufactured. The Affiblot, which is a portmanteau of “affinity” and “dot-blot,” is a palm-size device that is operated by a vacuum pump. The addition of reservoirs on the top of the lid and microfluidic drain channels at the bottom of the sample wells enabled us to dispense and drain reagents from the wells without the need for device disassembly. In comparison to the conventional dot-blot, the whole procedure on Affiblot could be finished on-device in a shorter time with less manual labor. Parallel screening of several samples was possible without the need to cut the membrane and treat each piece separately.

The Affiblot device helped with the evaluation and comparison of the affinity, cross-reactivity, and batch-to-batch variability of antibodies. While mostly used for polyclonal and monoclonal antibodies, proteins, and peptides, the device is suitable for whole-cell bacterial antigens as well.

Previously, the Affiblot device was fabricated by CNC (computer numerical control) milling from polycarbonate plates. This method uses computer-operated stepper motors to control the movement of a rotating cutting tool. The control code read by CNC software is generated based on a CAD blueprint. In our experience, however, problems with manufacturing reproducibility often occurred, among others, leakage of liquid or air in various parts of the device.

The Affiblot device introduced in this study was specifically designed for fabrication using stereolithographic (SLA) 3D printers. Although 3D printing is not yet widely adopted in microfluidics, the field is evolving rapidly. Historically, the complex manufacturing processes and specialized engineering tools posed significant barriers to the development and widespread adoption of microfluidic technologies. However, embracing the philosophy of “from cleanroom to makerspace,” the continuous advancements in 3D printing have democratized the involvement of end users, including chemists, biologists, and hobbyists in general, in the innovation of microfluidic devices, thereby enhancing the accessibility of this technology [11]. The potential of 3D printing in bio-applications and microfluidics lies in its automated nature, allowing for a single-step fabrication of intricate devices with integrated features such as valves, fluidic interconnects, interfaces, electrodes, and more [12]. The introduction of new printable materials, including metals, polymers, and ceramics, has expedited the rapid prototyping of tools applicable in biology and chemistry labs. In general, it is essential to consider the quality of prints and the cost of the machines, which both increase in the order of FDM < SLA < DLP < laser sintering < PolyJet < multi-photon printers [13]. Early applications of 3D printing in the early 2000s primarily focused on fabricating molds for PDMS microfluidic devices [14]. However, it was not until the late 2000s and early 2010s that 3D-printed microfluidic devices began to emerge [15–17], often utilizing the more precise PolyJet machines.

The stereolithographic 3D printing was first proposed by Chuck Hull who patented the concept for the SLA 3D printer in 1986 [18]. Stereolithography is a method of 3D printing that builds the products layer-by-layer using UV or near-UV light inside a tank of photocurable resin. The thickness of the printed layer is determined by the width of the gap between the bottom of the tank and the previously cured layer [19–21]. The utilization of SLA technology in microfluidics gained prominence in the mid-2010s [22–24], with SLA becoming increasingly popular for both constructing microfluidic devices and preparing molds for soft lithography [25–31]. Stereolithographic printers, even among relatively affordable commercial options, are now reaching resolutions suitable for microfluidic device production.

This paper serves as a follow-up to our previous work, highlighting advancements in design and technology, particularly showcasing a significantly less laborious and streamlined production process. Two variants of SLA technology were tested—the laser-SLA-based low force stereolithography (LFS) and masked SLA (MSLA). The original design employed a system of open microchannels, which was, at the time, the only possible solution compatible with the available CNC milling machine. The fourth wall of the channels had to be added in post-processing as a polypropylene (PP) foil pressed onto the channels by a rubber pad. This design also proved very conveniently compatible with the fabrication by 3D printing. In SLA printing, closed fluidic channels smaller than a certain diameter (about 1 mm) could become fused shut. The open channel design bypassed these issues, allowing us to print microchannels around 200 μm in diameter.

## Materials and methods

Human epididymal secretory protein HE4, anti-HE4 mouse monoclonal antibody (clones 2B13 and 3C24), all HyTest, Ltd., Turku, Finland; HRP-conjugated rabbit anti-mouse anti-IgG antibody, Sigma-Aldrich Corp., St. Louis, MO, USA; rabbit polyclonal anti-endoglin antibody H-300 (lot sc-20632), Santa Cruz Biotechnology, Inc., Dallas, TX, USA; bovine serum albumin (BSA), Sigma-Aldrich Corp., St. Louis, MO, USA; ClarityTM WB ECL Substrate (#1705060), Bio-Rad Laboratories, Hercules, CA, USA; washing buffer (PBS-T, PBS with 0.05% Tween 20), equilibration buffer (10 mM phosphate buffer, pH 7.3), blocking buffer (5% BSA in PBS-T), primary antibody buffer (0.25% BSA in PBS-T), all Penta, Chrudim, Czech Republic.

### Low force stereolithography (LFS)

In the laser SLA, the beam of a laser is reflected into the tank to selectively cure the resin. This research used the FormLabs Form3 printer (laser: 250 mW, 405 nm; XY resolution: 25 μm, laser spot size: 85 μm, layer thickness: 25–300 μm, build volume: 145 × 145 × 185 mm, automatic tank refill) by Formlabs, Inc., Somerville, MA, USA. In the case of this printer, the laser does not expose the resin in continual passes but by scanning the layer in short linear segments. The LFS technology advertised by the producer is characterized by a flexible bottom of the resin tank, which should reduce the forces required to peel off the cured layers.

The declared composition of FormLabs Standard Clear resin used with this printer is 55–75% urethane dimethacrylate, 15–25% methacrylate monomers, and <0.9% photoinitiator diphenyl (2,4,6-trimethyl benzoyl) phosphine oxide.

The CAD models for the printer were drafted in Autodesk Inventor and exported as STL files into the FormLabs PreForm 3D SW. The software is supplied with a library of preset printing conditions for each available resin. The high-detail components of Affiblot were printed at 25 μm layer thickness, the rest at 50 μm layer thickness.

### Masked SLA (MSLA)

The MSLA method uses a continual stationary source of light, and the selective exposure is provided by a photomask projected on an LCD placed over the light source.

The MSLA printer used for comparison with the LFS was the Anycubic Photon (UV-LED 405 nm, masking by 2560×1440 LCD, XY resolution: 43 μm, layer thickness: 25–100 μm, build volume: 115 × 65 × 155 mm) by Shenzhen Anycubic Technology Co., Ltd., Shenzhen, Guangdong Province, China.

The composition of Anycubic Resin Clear/Green declared by the producer is 30–60% polyurethane acrylate, 10–40% acrylate monomer, and 2–5% photoinitiator.

The STL models were imported into Photon Workshop SW. Because there was no resin library, the curing conditions had to be set manually. Again, the high-detail components were printed at a minimum layer thickness of 25 μm, and the rest at 50 μm.

### Computer numerical control (CNC) machining

The CNC machine used in this research was relatively affordable build (circa 3500 USD) by KB Electronics, Hostomice, Czech Republic. The machine was equipped with a 1.5 kW spindle (max. 24 000 RPM) and 150 N cm^−1^ stepper motors (200 steps/revolution), with stepper drivers capable of splitting the steps into 8 micro-steps. The automatic step-per-unit (SPU) setting for the CNC software thus showed 320 steps/mm. The precision declared by the producer was 0.05 mm.

Affiblot was mainly machined from PALSUN polycarbonate (PC; Supplementary Information, Fig. S7) and/or polyoxymethylene (POM, also polyacetal), specifically a POM copolymer sold commercially under the name Ertacetal.

### Affiblot

The Affiblot device was designed upon the commercially available dot-blot devices. The device was assembled from three main components (SI, Fig. S5). The sample template, which served as the lid, contained on the top part of the lid an array of 5-by-5 sample wells; each row of five wells was supplemented by one reservoir for 1 mL of liquid. The drainage channel system was added to the bottom side of the lid that was connected to a vacuum output.

The drainage system was composed of open microchannels (200 μm wide and 50 μm deep), which connected the sample wells to the larger open collector channels (1 mm wide, 500 μm deep). To add the fourth wall to the open drain system and to shield the membrane, a fitted PP foil was snapped on top of the membrane using four slotted pins.

**Fig. 1:**
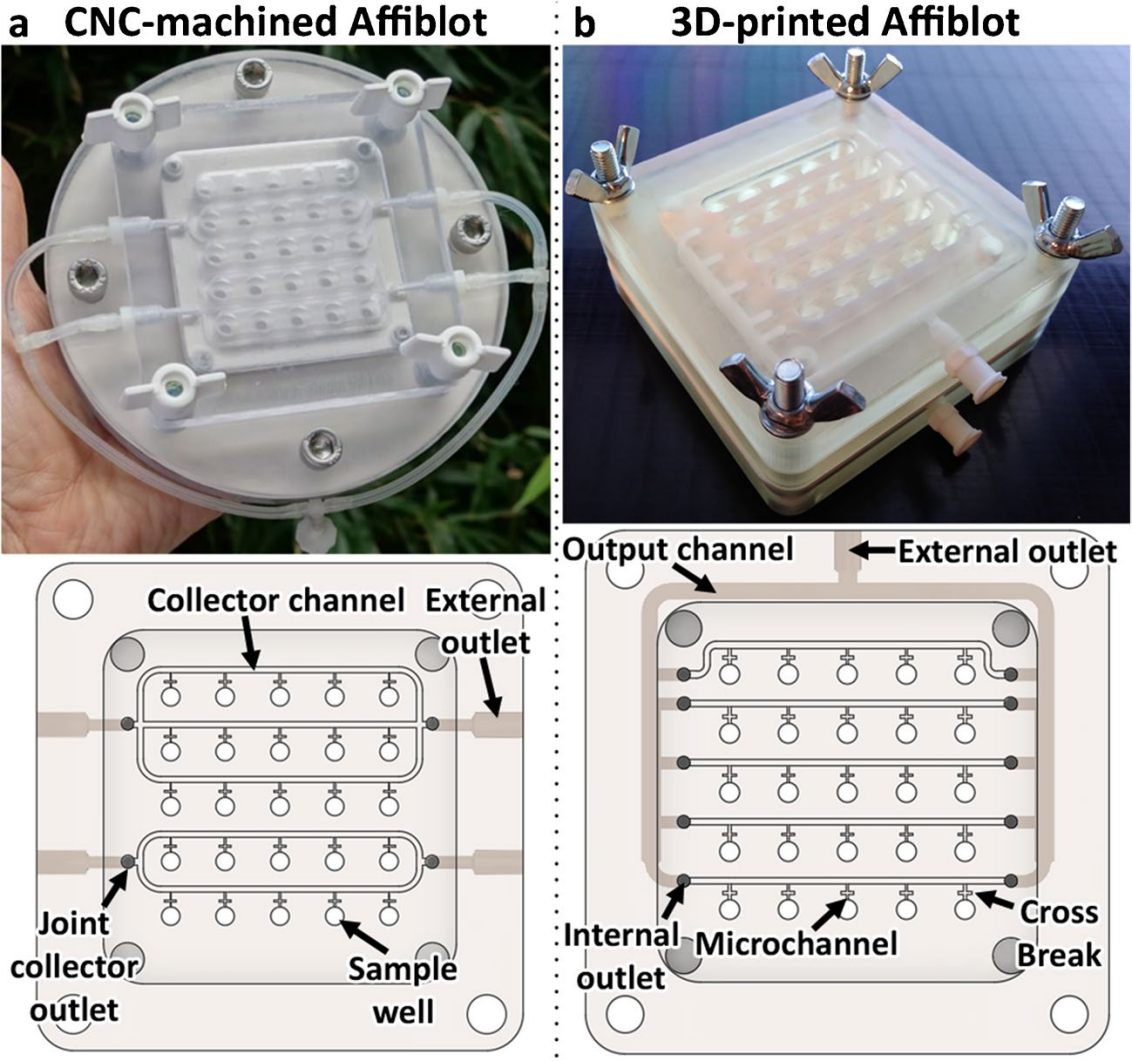
Comparison of the CNC-milled and the 3D-printed version: **a** the impossibility of fabrication of internal channels by CNC milling led to the necessity of the 3-and-2 joint collector design connected to external output tubing; **b** 3D printing allowed individual collection from each row into an internal output channel, the overall size of the device was reduced for 3D printing to accommodate the build platform size limitation and to economize resin consumption

The liquid was drained from the wells through the open microchannels to the collector channels and then to the output channels. In the 3D printed prototype, these channels could be fabricated inside of the device, while the milled prototype had to be provided with external tubes connected to the external outlets with vacuum output (Fig. 1).

Among the remaining components was the support plate with a platform that housed the membrane with one replaceable PP foil on the top and on the bottom that allowed support and protection of the membrane from the liquid and force of the vacuum. A shape-fitted porous-silicone gasket pressed a blotting membrane and the foil cover against the drainage system. The last component was a vacuum manifold with a vacuum chamber for sample deposition and vacuum output (SI, Fig. S6).

Four hex-head screws were embedded in hexagonal counterbore slots in the bottom plate of the device, passing upwards through all three device components. The components were then tightened with wingnuts. To accommodate for stress damage potentially caused by the tightened screws, the surroundings of the screw holes were thickened in later iterations of the device (SI, Fig. S7c).

The antigens in the sample wells were applied on the blotting membrane using a vacuum connected to the vacuum chamber underneath. The antigen dots created this way were then processed by the addition of reagents such as primary and secondary antibodies and washing buffer to the wells. The liquid did not drain spontaneously into the drain channels, which enabled on-device incubation. The fluids could then be easily removed from the device by applying a vacuum to the system of the drain channels in the lid. That way, the blotting technique could be performed fully on-device.

### Functionality testing

The functionality of the device was tested by performing a 5-point calibration experiment with an HE4/anti-HE4-antibody system. Two anti-HE4 antibodies, both in two dilutions, were incubated on-device with increasing concentrations of HE4 antigen.

HE4 is a biomarker for malignant gynecological cancer, such as ovarian cancer, with high potential in future oncological diagnostics and research and one of the promising analytes in our future research. Both the monoclonal anti-HE4 antibodies (2B13 and 3C24) were purchased from HyTest, Ltd., Turku, Finland. Two working solutions of both antibody clones were prepared by diluting the stock solution 1:1000 and 1:3000 with PBS. The HE4 antigen was deposited onto the membrane using a vacuum in four rows onto the 5-by-5 array. In column 1, 1 ng of HE4 was applied, in column 2 was 10 ng, in column 3 was 50 ng, and in column 4 was 200 ng of HE4. In column 5, as well as the whole row 5 were zero ng HE4 and those spots served as blank. The membrane was blocked by incubation with 5% BSA in PBS-T for 1 h. Then, the antibody solutions were added into the thoroughly washed wells with PBS-T: in row 1 the antibody 2B13 diluted 1:1000 was added, in row 2 the antibody 2B13 diluted 1:3000, in row 3 was added the antibody 3C24 diluted 1:1000, and in row 4 the antibody 3C24 diluted 1:3000. Row 5 was again left as a blank and filled by PBS instead of the antibody. After on-device incubation and thorough washing by PBS-T through the drain system, an HRP-conjugated secondary anti-IgG antibody was added to all wells and again incubated and washed. The immune complexes of HE4/antibody were detected by the ChemiDoc™ imaging system (BioRad) using Clarity™ WB ECL substrate, a western blot visualization kit containing peroxide reagent and luminol reagent.

## Results and discussion

Three fabrication methods were tested in the development of the Affiblot—CNC machining, MSLA printing, and LFS printing. Each of these methods provides its own set of advantages and disadvantages, and differences in price, resolution, and post-processing are discussed below.

### CNC machining v. 3D printing

The first method used for Affiblot fabrication was CNC machining. This method was relatively time-consuming, with multiple manual post-processing steps, which was one of the major contributors to the extension of the fabrication time in CNC milling. The architecture of our CNC machine enabled only 2D and 2.5D (3D sculpting in layers of 2D contours) milling. This meant that all double-sided components and components with machinable features on their side required either extensive alignments for the CNC or being machined manually. Although CNC milling was faster than 3D printing, the cutting tools often left burrs along the milled edges of the microfluidic channels that have to be carefully manually removed. If not, these burrs would often obstruct the drain system. This manual deburring and refinement of the whole system of drain channels took hours, even days, and significantly prolonged the manufacturing time. The manufacturing irregularities and the manual post-processing would also often compromise the functionality of the drain system. Therefore, frequent leakages or irregular draining occurred and led to lower reproducibility of the manufacturing process and a higher rate of failed production pieces.

On the other hand, post-processing in SLA 3D printing proved significantly less demanding. It involved mostly washing in isopropanol and post-curing under UV to finalize the curing of the photoresin.

A major positive of 3D printing compared to CNC milling is the possibility of fabricating hollow structures, such as cavities and channels. These have to be designed in a manner that avoids the trapping of the uncured resin. Partial curing and structural fidelity of the printed features have to be also accounted for; otherwise, it can lead to the fusing and loss of very small internal structures.

CNC milling does not allow the fabrication of internal channels; therefore, the Affiblot microchannel system had to be drained through four outlets manually drilled to the side of the sample template plate (Fig. 1a). The number of these threaded outlets was dictated by the size limitations of the template plate and the need to reduce the extent of manual post-processing. These outlets were connected through external silicone tubing to the vacuum pump. On the contrary, 3D printing allows the fabrication of internal structures. Each of the open collector channels could be drained by its own set of 10 internal outlets. These could continue as 2-mm-wide internal channels and connect to a single external outlet (Fig. 1b).

A major contrast between CNC machining and 3D printing arises from the difference in the fundamental operations of subtractive and additive manufacturing. In CNC machining, products are cut out of a pre-existing block of solid material. 3D printing, on the other hand, shapes products by building them up, adding one thin layer of the material after another [32]. This difference in operations is projected into material loss and waste. Comparing the price, 1 dm^3^ of higher quality polycarbonate would cost roughly 15 USD, which would make SLA resins (priced at about 150 USD per liter) 10× more expensive per volume. Despite that, 3D printing converts most, if not all, of the raw material into the product. In milling, the removed material is discarded as waste. Unoptimized workpiece setup in CNC machining could lead to over 50% of material losses to shavings and unsalvageable cutoffs and frames.

Another aspect for consideration is the consumption of electricity, which is much higher in machining. While the power input of the 3D printer was 150 W, the available CNC machine required 2.2 kW. The additional machining cost also includes the consumption of cooling or lubricating fluids. Considering all factors, the overall operation costs of CNC machining vastly exceed 3D printing and would significantly contribute to the final price [33–35].

The possibility of production almost without waste materials and the low energy consumption open the point of view of sustainability. The 2 kW difference in power input represents an order of magnitude difference in energy consumption and, thus, in the environmental burden. Furthermore, because waste generation is significantly reduced, the environmental impact of 3D printing is improved even further.

The economic point of view of the production with both of the technologies was analyzed in much more detail. The data was collected from time study analysis reports performed during the Affiblot device fabrication using both 3D printing and CNC milling. All types of costs included in direct costs of production, such as material costs, labor costs, depreciation of production equipment, and energy costs, have been identified. When calculating these costs, the total volume of manufactured items and the amount of investment in the acquisition of production equipment played a role. For 3D printing, direct production costs averaged 76 USD per piece, while the production by CNC milling resulted in an average cost of 236.55 USD per piece. In the comparison of individual types of costs, although CNC had lower material costs, due to the higher proportion of manual work, labor costs were significantly higher. In terms of investment costs, CNC milling equipment was cheaper, but direct production costs were much cheaper for 3D printing. In addition, the cost-effectiveness of 3D printing would increase as production volume increases (SI, Table ST2).

### MSLA vs. LFS 3D printing

**Fig. 2:**
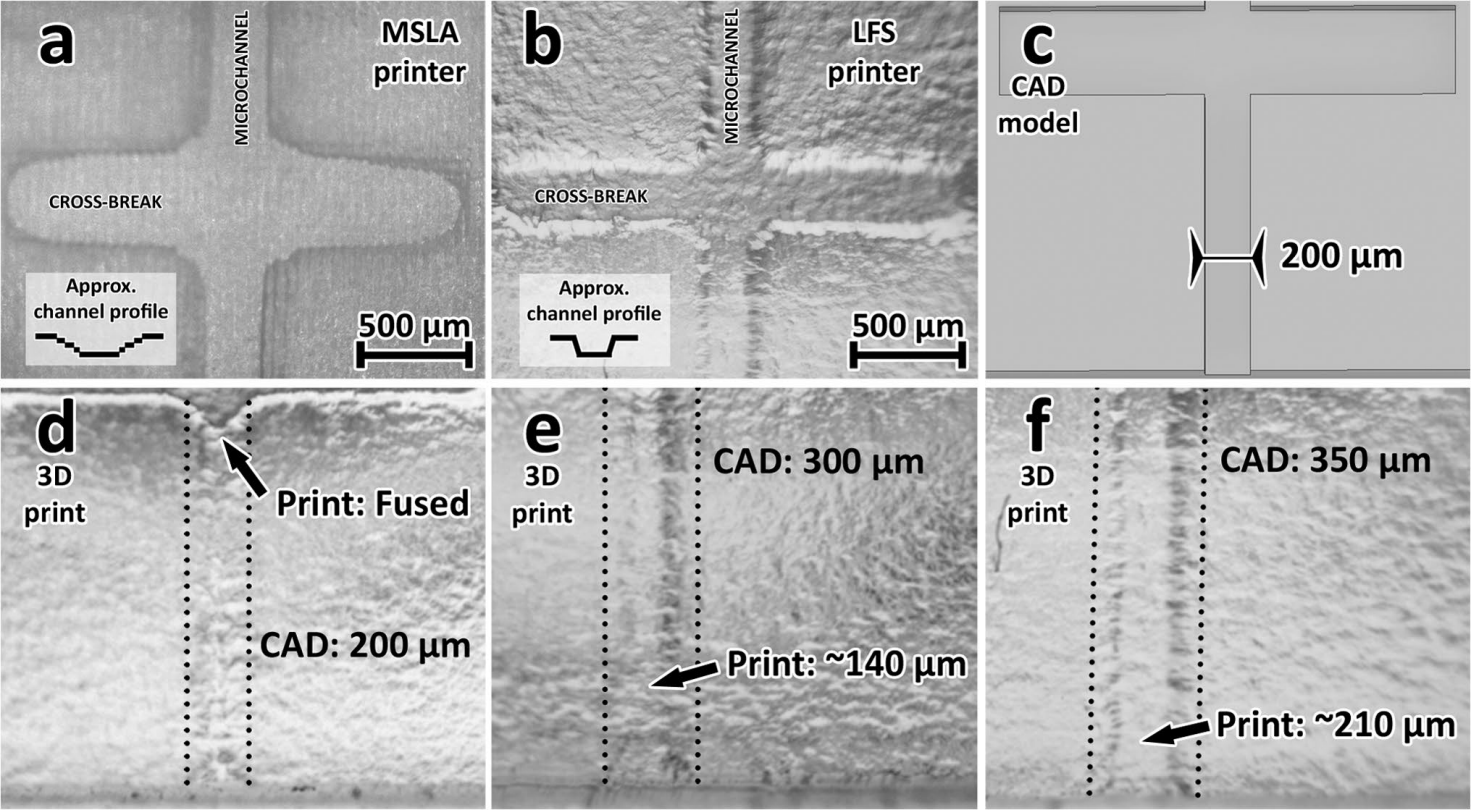
**a** The MSLA-printed walls had a very gradual profile, which led to severe leakage all across the drain system; **b** the LFS-printed channels shrank during curing but sufficiently retained the liquid; **c–f** LFS prints of the microchannels of different widths; **c** the original CAD model with a channel width specification of 200 μm; **d** product printed from the model in image **c**; the channel walls fused, which blocked the channel off; **e** product printed from CAD files with a channel width specification of 300 μm; although not as well-defined, the channel was operational; **f** the product printed from CAD files with a channel width specification of 350 μm; the channel width of this print finally roughly reached 200 μm

Initially, an Anycubic Photon (retailed at roughly 300$) was intended for Affiblot fabrication, but the small 115 × 65 mm build platform and lower resolution impeded the successful adaptation. While the total dimensions of the Affiblot could be reduced to (very barely) fit onto the printing platform without intervening with the design and dimensions of the microfluidic drainage system, the size reduction was to the detriment of the comfortable manipulation and durability. The printing of 300-μm-wide, 50-μm deep open microchannels proved to be beyond the capability of the resolution of this printer. While the open microchannels did get printed, the lower resolution and blurring through indirect exposure caused the production of poorly defined walls with a gradual incline instead of a sharp right-angle partition (Fig. 2a). This caused issues with the tightness of the microchannels and overall leakage of the product.

The other tested printer was FormLab Form 3 (retailed at roughly 4000 USD). The printer uses laser SLA technology, specifically so-called low force stereolithography, which utilizes a flexible resin tank. This should facilitate the easier release of the cured layers from the tank bottom. Reduced release stress supported the utilization of smaller, less-dense supports for printing, and, thus, a reduction of touch marks on the product’s surface [36]. The 145 × 145 mm build platform of the Form 3 printer did not require any alterations to the original dimensions of the Affiblot.

Internal channels under 1 mm in diameter are prone to trapping uncured resin or fusing. The finer conditions that are required for the fabrication of smaller channels usually demand the utilization of custom-built printers and/or custom resin mixtures [37]. Because any closed sub-millimeter channels would be very difficult to print on a commercial printer [38], the open channel system that we developed for the previous iteration of the device was much more compatible with 3D printing. The open channels printed in the LFS printer were much better defined compared to the ones printed on the MSLA printer and could thus be sealed properly without leaks (Fig. 2b).

Although it was possible to print very small open channels, the width of about 200 μm proved to be limiting even for the LFS printer. The inaccuracy of printing was likely caused by the diameter of the curing laser beam, indirect exposure, and the printing resolution in general, which led to the curing of excess material around the walls of the channel. This resulted in an addition of 70–80 μm of material to each wall of the channel and a significant narrowing of the width of the channel. This led to the blockage of channels below a certain width. Therefore, this loss of width had to be accounted for during CAD drafting. Figure 2d–f shows a comparison of microscope photographs of 3D-printed microchannels originally designed (in the respective STL files) as 200 μm, 300 μm, and 350 μm wide, all set at a depth of 50 μm. The channels designed at the width of 200 μm ended up with their walls fused (detail in SI, Fig. S3 and S9), and only STLs with channel sizes set above 300 μm fulfilled their roles without issue.

### Prototyping of the drain system

The addition of the microfluidic drainage to the lid of the device allowed the crucial option of on-device sample processing, which is not possible in regular dot-blot devices. The microfluidic drainage system, which was active only when applying a vacuum to the upper external outlet (EO), did not exhibit any communication between sample wells, so-called cross-talk. Because of the isolation of the wells, each sample could be potentially exposed to different reagents or concentrations of reagents.

**Fig. 3:**
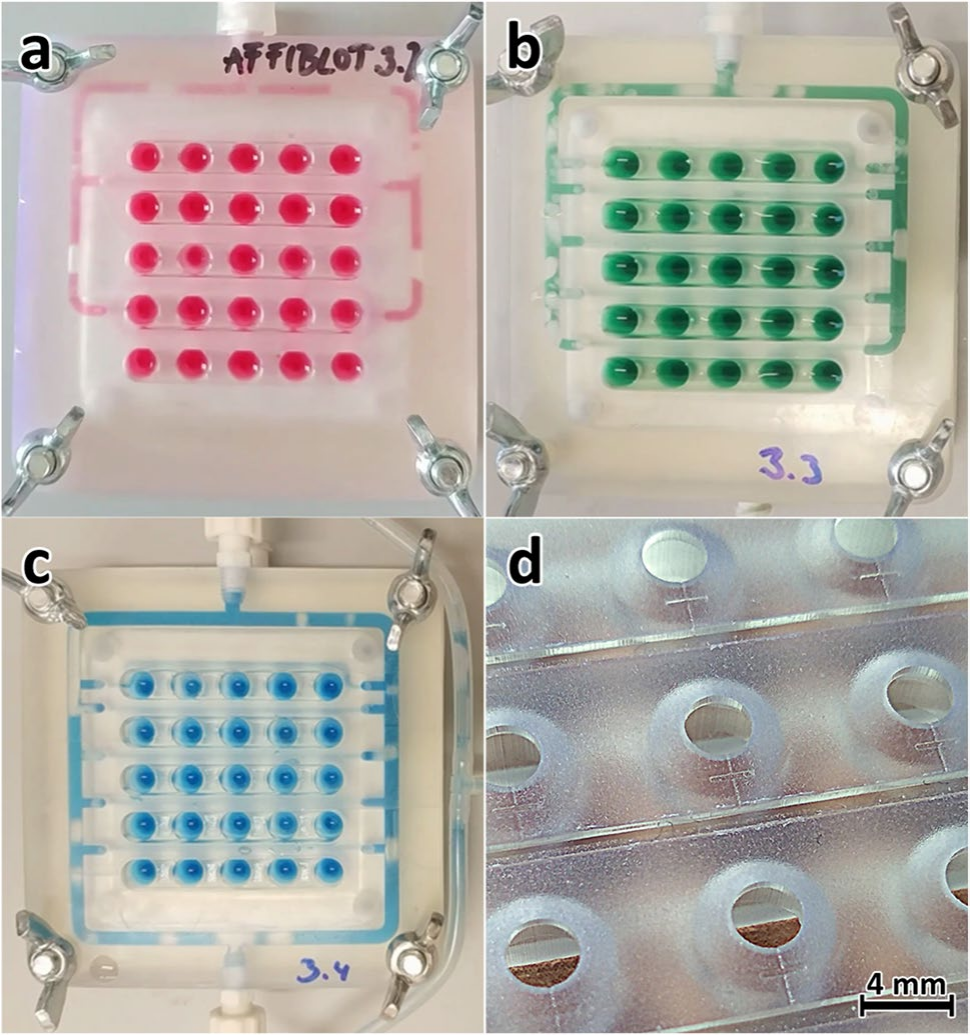
Prototyping of the designs of the drain system; **a** the 3-and-2-row joint collector outlet (remnant of the CNC-milled design) with an internal output channel (setup: 4 IO, 1 EO); **b** individual collector channel outlets for reduced risk of cross-contamination (setup: 10 IO, 1 EO); **c** second external outlet to balance the vacuum applied to each well (setup: 10 IO, 2 EO); **d** detail of the 3D-printed drainage wells and micro- and collector channels

As mentioned before, the initial design of the drain system was limited by the prospect of manual post-processing after CNC machining. The collector channels had to be joined into joint internal outlets (IO) to reduce the number of holes manually drilled and threaded into the side of the device. This concern disappeared with the addition of internal output channels in 3D printing. Not only that, the robustness of the fabrication method also allowed for very fast prototyping of the design. While the first 3D-printed prototypes still used the 3-and-2 joint collector design (Fig. 3a), in the next prototype, each of the collector channels drained individually into the internal output channel (Figs. 1b and 3b; detail in SI, Fig. S7). This was meant to further reduce any risk of cross-contamination between the sample wells and improve the vacuum distribution in the system. Another attempted redesign was the addition of a second external outlet to help further balance the distribution of vacuum between all of the wells (Fig. 3c).

Splitting the collector channels into ten individual internal collector outlets (3b) instead of four joint internal outlets (3a) halved the drain time from 28 to 14 s. The initial tests comparing the single external outlet (3b) and the double external outlet (3c) versions revealed that adding the second external outlet did not improve vacuum distribution or the drain rate. On the contrary, part of the liquid drained into the output channel of the double outlet version, and a portion of it often started oscillating back and forth between the two external outlets instead of being drained out of the device.

Cross-talk between the sample wells through the drainage system was tested by alternating application of the antigen and blank solutions into the wells (SI, Fig. S4). After performing complete standard sample processing and visualization procedure, the membrane showed no color reaction in the blank spots surrounding the visualized spots of the antigen. This confirmed that no communication between the wells was occurring at any time during the procedure.

**Fig. 4:**
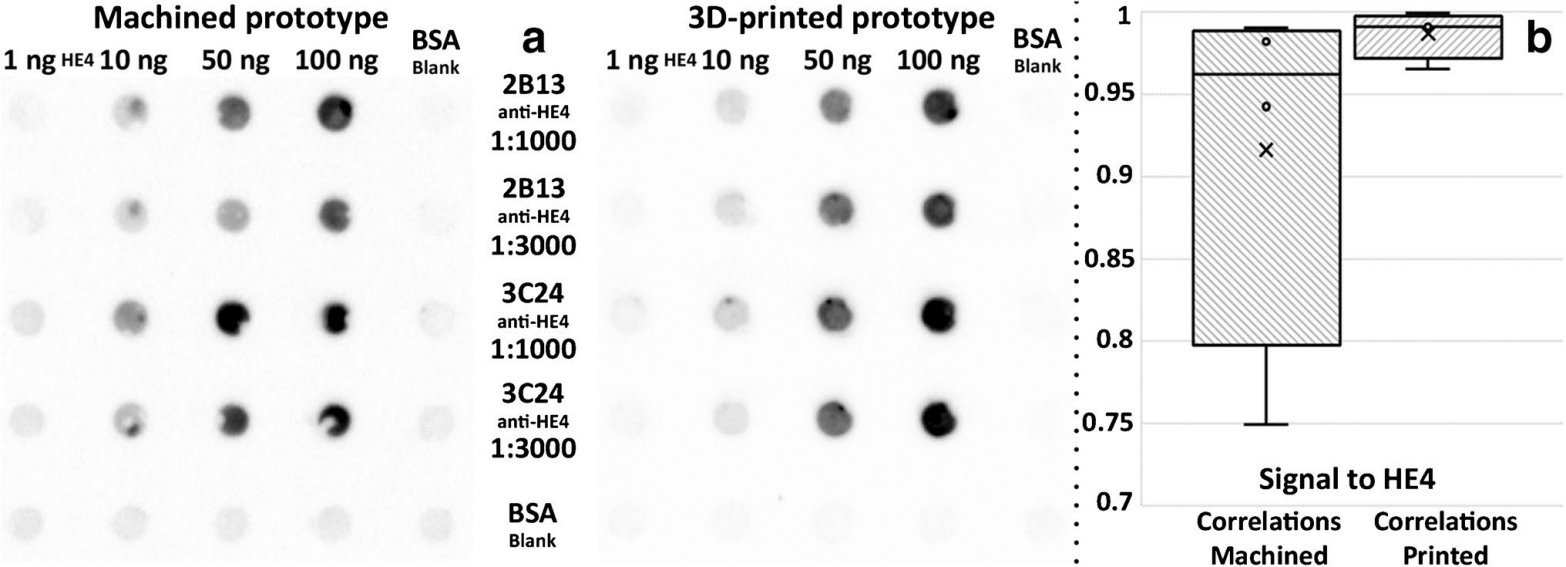
**a** Comparison of the previous CNC-micromachined functionality and the new 3D-printed Affiblot device; an identical calibration procedure was performed on both devices simultaneously; spots of an HE4 antigen were deposited onto the membrane, and two dilutions of two different clones of anti-HE4 antibodies were added to form the immune complexes. **b** Box charts of the correlations of colorimetric signals to the concentration of the HE4 antigen in the CNC-machined and the 3D-printed device

The functionality of the 3D-printed device was compared to the CNC-machined version reported in the previous paper [10]. The identical 5-point calibration experiment was performed on both devices concurrently. The two available anti-HE4 antibody clones 2B13 and 3C24 were added in both the 1:1000 and the 1:3000 dilutions to the HE4 antigen, which was deposited onto the membrane in increasing concentrations from 1 to 100 ng per well (see Fig. 4a; SI, Fig. S1).

The intensity of the visualized spots was measured, and increasing intensity for both antibody dilutions was compared (SI, Fig. S2, Table ST1). The antibody 2B13 showed slightly higher intensity in 1:1000 compared to 1:3000 on the CNC-machined device as expected. The same results were obtained for the antibody 3C24. The 3D-printed device provided more consistent data; the intensity in spots of 1:1000 and 1:3000 diluted antibodies were almost identical. From the antibody point of view, the dilution 1:3000 can be used for both antibodies without significant loss of the signal. Both devices provided the same data tendency, with datasets from devices showing Pearson’s correlation *R* at 0.9484 and *p*-value <0.001. Yet, the 3D-printed device showed better correlation and linearity of colorimetric signal response to the concentration of the deposited antigen (Fig. 4a). The experiment demonstrated that the 3D-printed device can operate with complete functionality, outperforming the CNC-machined counterpart.

## Conclusions

Switching from CNC machining to 3D printing allowed us to streamline the production of the Affiblot device. We did not take notice of any issues with the performance of the 3D-printed device compared to the previous CNC-micromachined device, indicating no loss of functionality. On the contrary, 3D printing facilitated a faster prototyping process and better reproducibility, which reduced the time between the model design and the tangible product prototype.

Although the build materials for 3D printing are more expensive per piece, much higher required man-hours greatly outweigh the amount of material waste generated by CNC milling, higher energy consumption, and other miscellaneous operation costs, such as cooling fluids. Considering all of the factors, the calculations of the production cost analysis showed about 2.5–5× higher total per-piece cost in CNC milling than in 3D printing. The lower energy consumption and reduced waste, hand in hand with the easy replacement of lost or broken parts of the device by 3D printing, improve the environmental impact of 3D printing.

The drain microchannels of the Affiblot had to be designed at the width of 300 μm in the 3D model to compensate for the loss of detail between the CAD model and the 3D-printed product, which resulted in the anticipated width of roughly 200 μm on the printed product. Moreover, the fabrication method is highly user-friendly and requires almost no additional manual retouching or processing, such as time and labor demanding manual deburring of the microchannels, compared to CNC milling. 3D printing also opens a possibility for inter-institutional collaborations and technology sharing by simply providing CAD files for the components.

Printing of closed microfluidic channels below 1 mm in diameter may still be difficult outside the in-house build custom printers often used in research. We circumvented the problem by printing our microchannels as open structures and completing them with a removable fourth wall made of PP foil. This way, we could 3D print a device with 200–300 μm by 50 μm microchannels on a readily available commercial 3D printer.

### Supplementary Information

ESM 1: (PDF 1340 kb)
