# Supplementary material for: Advantages of stereolithographic 3D printing in the fabrication of the Affiblot device for dot-blot assays
Source: Mikrochim Acta. 2024 Jul 2;191(8):442. doi: 10.1007/s00604-024-06512-z (PMC11219379; doi:10.1007/s00604-024-06512-z)
Supplement: Supplementary file 1 — (PDF 1340 kb) [file 604_2024_6512_MOESM1_ESM.pdf]

Supplementary Information

Advantages of Stereolithographic 3D Printing in the Fabrication of the Affiblot Device

Jakub Novotny[1], Zuzana Svobodova[2], Marie Ilicova[2], Dominika Hruskova[3], Jana Kostalova[3], Zuzana Bilkova[4], Frantisek Foret[1]

[1] Institute of Analytical Chemistry of the CAS, v. v. i., Brno, Czech Republic
[2] Department of Biological and Medical Sciences, Faculty of Pharmacy in Hradec Kralove, Charles University, Czech Republic
[3] Department of Economy and Management of Chemical and Foodstuff Industry, Faculty of Chemical Technology, University of Pardubice, Pardubice, Czech Republic
[4] Department of Biological and Biochemical Sciences, Faculty of Chemical Technology, University of Pardubice, Pardubice, Czech Republic

Contents

1. Comparison of the CNC-milled and the 3D-printed Affiblot device 2
2. Profilometric analysis 4
3. Cross-talk testing 5
4. Images of the 3D-printed device 6

1. Comparison of the CNC-milled and the 3D-printed Affiblot device

Monoclonal anti-HE4 antibodies (2B13 and 3C24) were used to test the functionality of both the CNC-milled and the 3D-printed Affiblot. Two working solutions of both antibody clones were prepared by diluting the stock solution 1:1000 and 1:3000 with PBS. The HE4 antigen was deposited onto the membrane using a vacuum. The 5-by-5 sample spots were prepared according to the following diagram (**Figure S1**):

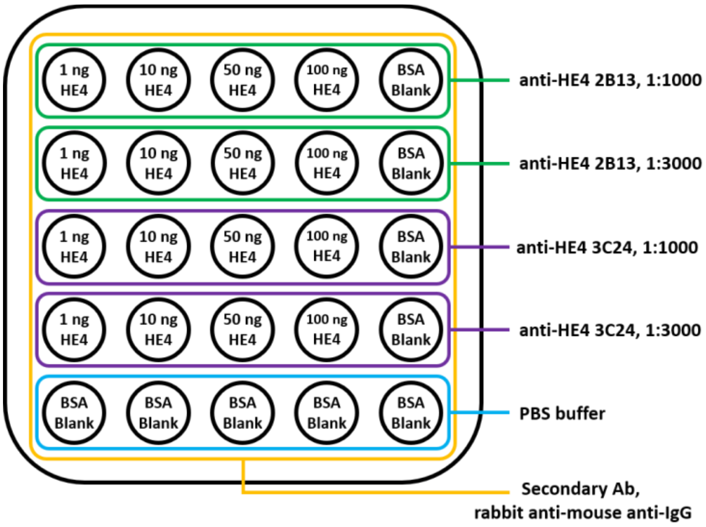

Figure S1. Diagram of the reagent deposition for the functionality test.

After on-device incubation and thorough washing, an HRP-conjugated secondary anti-IgG antibody was added to all wells. The immune complexes of HE4/antibody were detected by the ChemiDocTM imaging system (BioRad) using ClarityTM WB ECL Substrate.

Table ST1. Colorimetric signals measured on membranes from both Affiblot devices. 2 clones of monoclonal anti-HE4 antibodies (2B13 and 3C24) in 2 dilutions were added to the HE4 (human epididymal secretory protein) antigen

		HE4 (ng)			
		**1**	**10**	**50**	**100**
**CNC**	**2B13 Ab, 1:1000**	242 972	1 173 752	2 904 888	4 712 272
	**2B13 Ab, 1:3000**	273 128	975 376	1 543 128	3 117 700
	**3C24 Ab, 1:1000**	542 688	2 063 752	5 086 260	4 006 264
	**3C24 Ab, 1:3000**	426 836	1 467 096	3 099 232	3 779 812
**3D**	**2B13 Ab, 1:1000**	311 068	819 888	2 850 948	5 222 388
	**2B13 Ab, 1:3000**	218 836	811 408	3 577 248	4 643 484
	**3C24 Ab, 1:1000**	407 488	1 227 788	4 292 688	6 637 944
	**3C24 Ab, 1:3000**	240 920	716 048	3 975 092	6 306 352

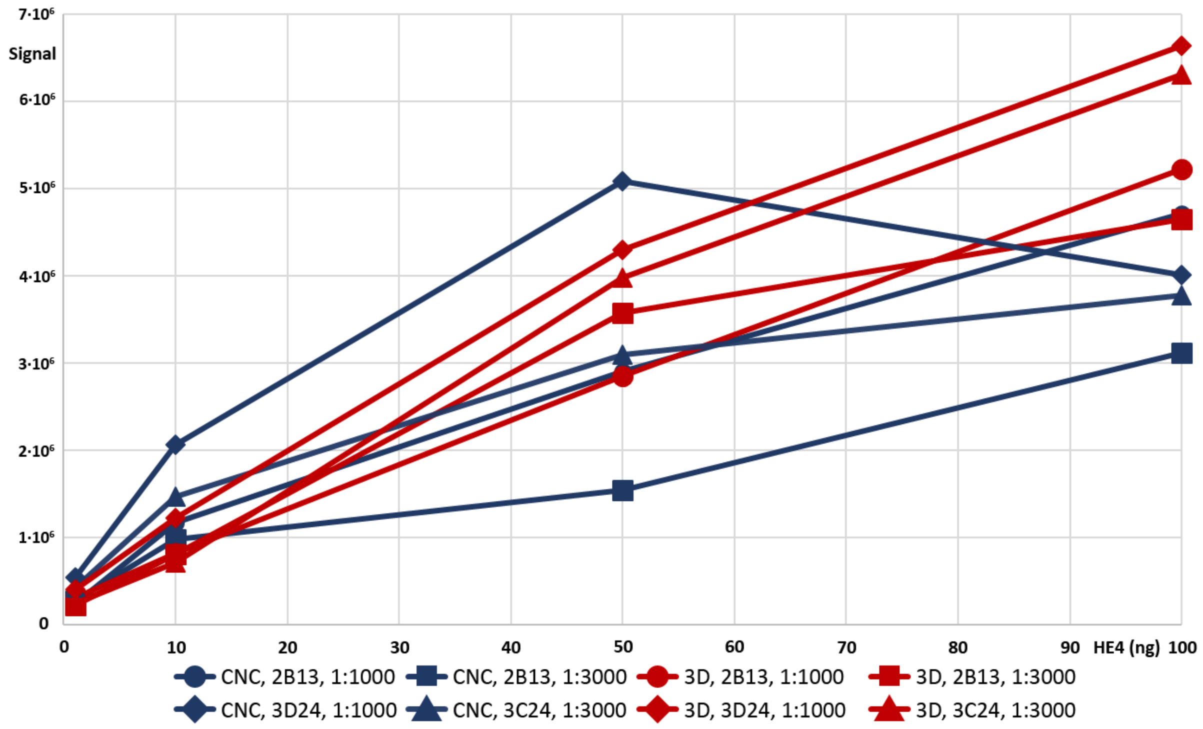

Figure S2. Plotted dependence of the signal on the HE4 concentration for the 2 anti-HE4 Ab clones, both at 2 dilutions. Blue plot lines represent measurements from the CNC-milled Affiblot, the red plot lines from the 3D-printed Affiblot.

Table ST2. Projected per-piece-cost decrease in a hypothetical upscale in commercial production. The listed amounts are based on the costs of equipment and average wages of workers and technicians in corresponding fields of expertise in the 2023 Czech Republic.

	**1 piece**	**10 pc./month**	**15 pc./month**	**40 pc./month**	**60 pc./month**
		**Single-shift operation**	**Single-shift operation**	**Double-shift operation**	**Double-shift operation**
**Materials**	32.76 USD	327.63 USD	491.45 USD	1310.55 USD	1965.82 USD
**Wages**	33.13 USD	331.23 USD	496.87 USD	610.44 USD	915. 67 USD
**Equipment – Annual/monthly depreciation**	2123.61 USD	176.97 USD	176.97 USD	176.97 USD	176.97 USD
**Energy consumption**	0.29 USD	2.89 USD	4.33 USD	11.54 USD	17.31 USD
**Total per piece**	**2189.79 USD**	**83.87 USD**	**77.97 USD**	**52.74 USD**	**51.26 USD**
**Pieces per year**	1	120	180	480	720

2. Profilometric analysis

The channel profile was characterized using KLA-Tencor Alpha-Step D-120 stylus profiler. Channels were 3D-printed in 2 widths – 200 and 350 microns. Comparison was made between the LFS 3D printer and the less expensive MSLA printer.

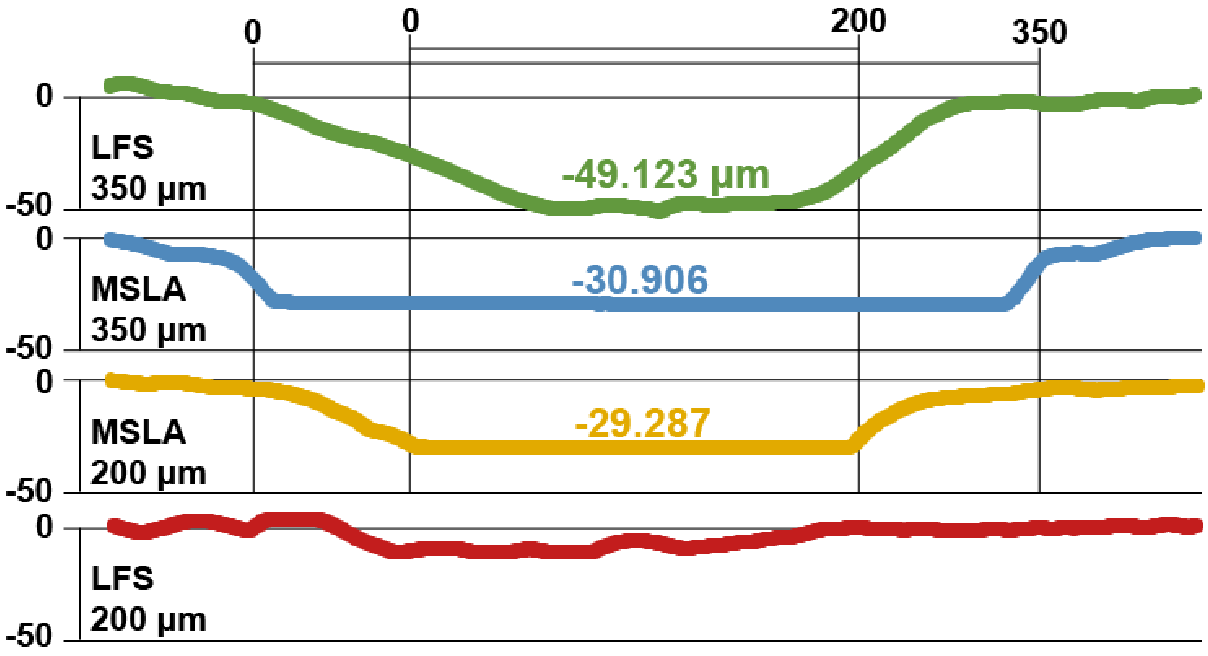

Figure S3. Comparison of profilometer scans of the microchannels printed by the MSLA and LFS printers. The 200-micron version of the LFS-printed channel fused during printing and the profile scan indicated only irregular flat surface. It should be noted that the channels printed by the MSLA printer could not be sealed properly and were prone to leaking.

3. Cross-talk testing

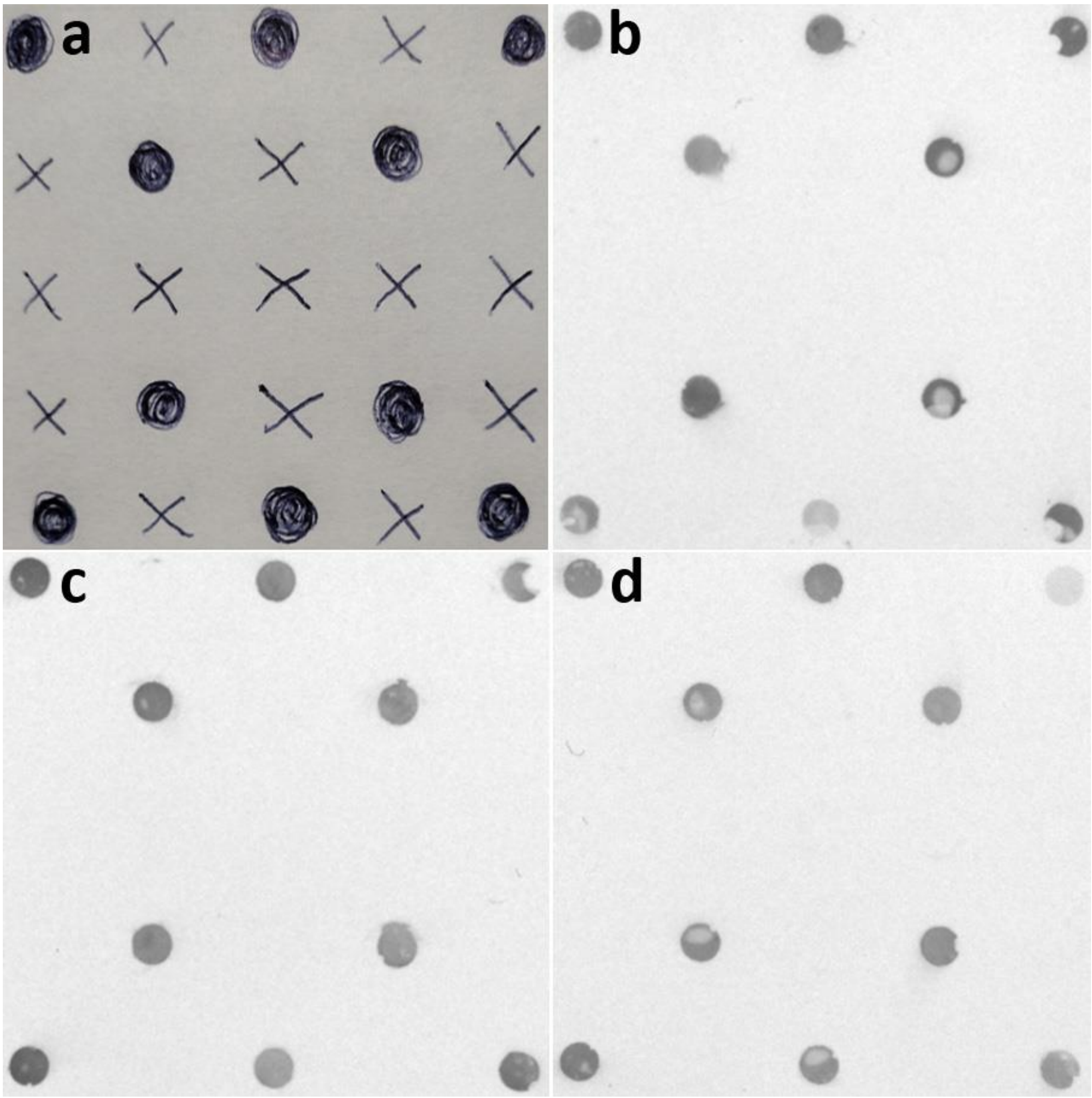

Figure S4. Cross-talk tests of the 3D-printed device involved the alternating application of the sample and blank solution into the sample wells (diagram in the top left, image a). Processed membranes (images b-d) were checked for leaks and signs of communication between wells. The sample used in the tests was an anti-endoglin antibody and was visualized by a secondary anti-IgG antibody and the Opti-4CN kit.

4. Images of the 3D-printed device

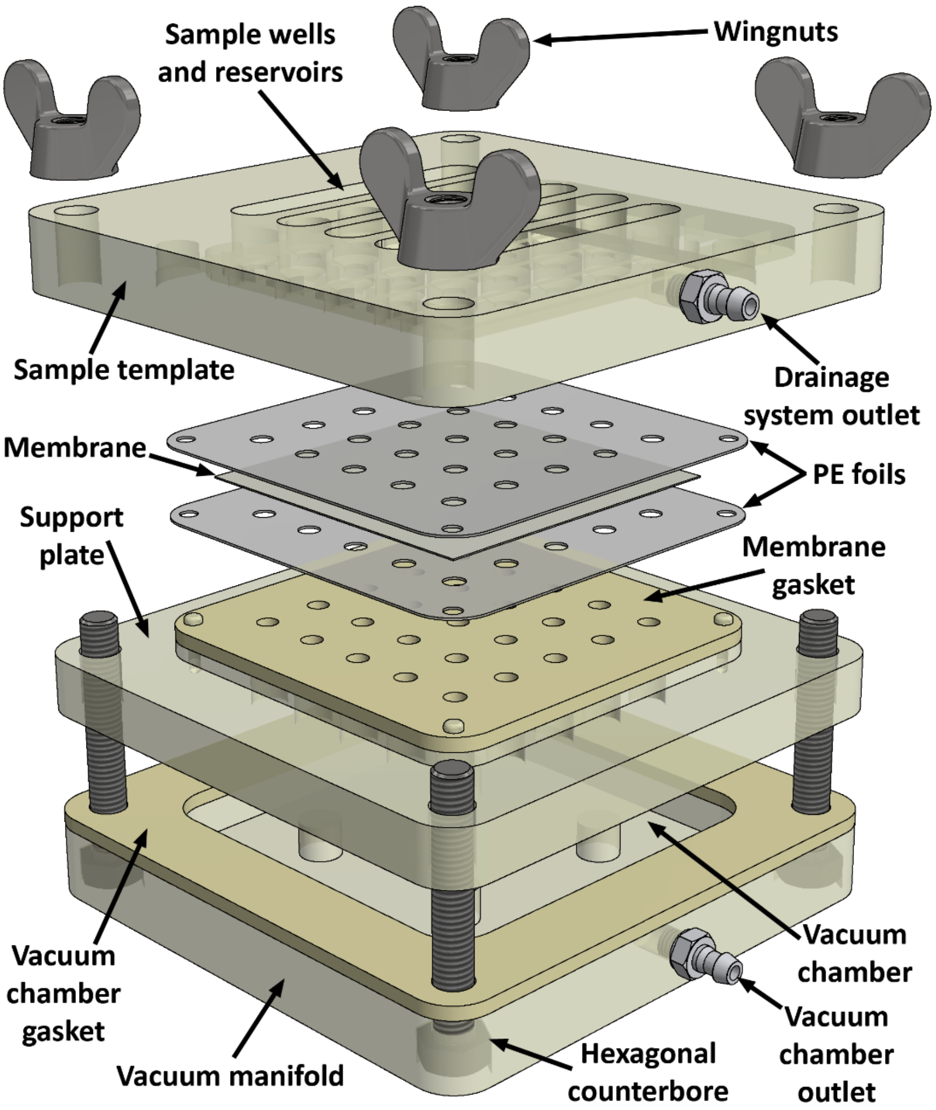

Figure S5. 3D-printed Affiblot. CAD model with the component list.

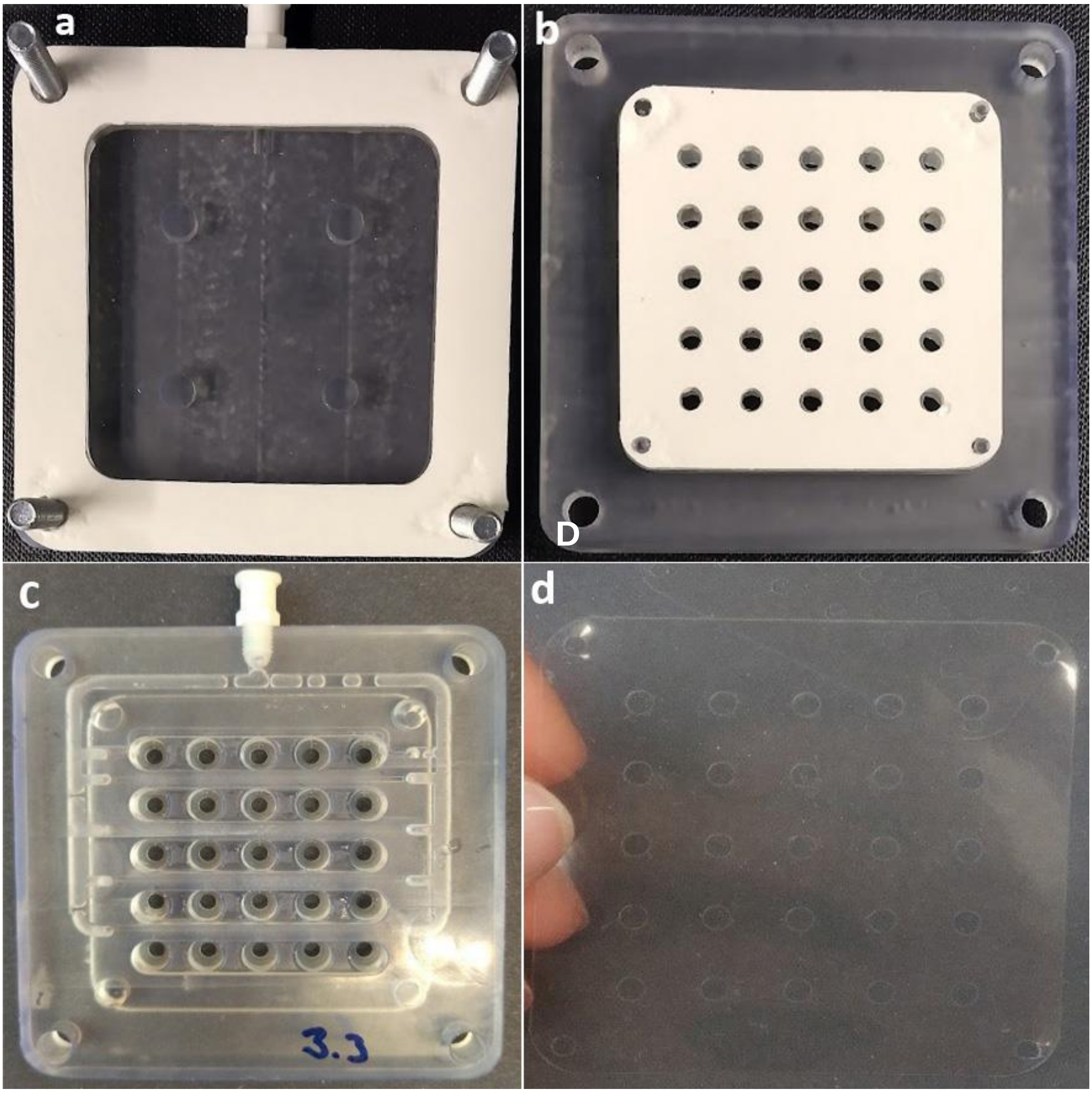

Figure S6. a: Bottom plate with the vacuum chamber. **b:** Middle supporting plate with the membrane platform and the shape-fitted gasket. **c:** The lid (10 IO 1 EO setup), or the sample manifold with the reservoirs, sample wells, and the internal portion of the drain system visible. **d:** Shape-fitted PP foils. The membrane is placed between the two foils which serve as the fourth wall of the open-channel drain system and as the support of the membrane.

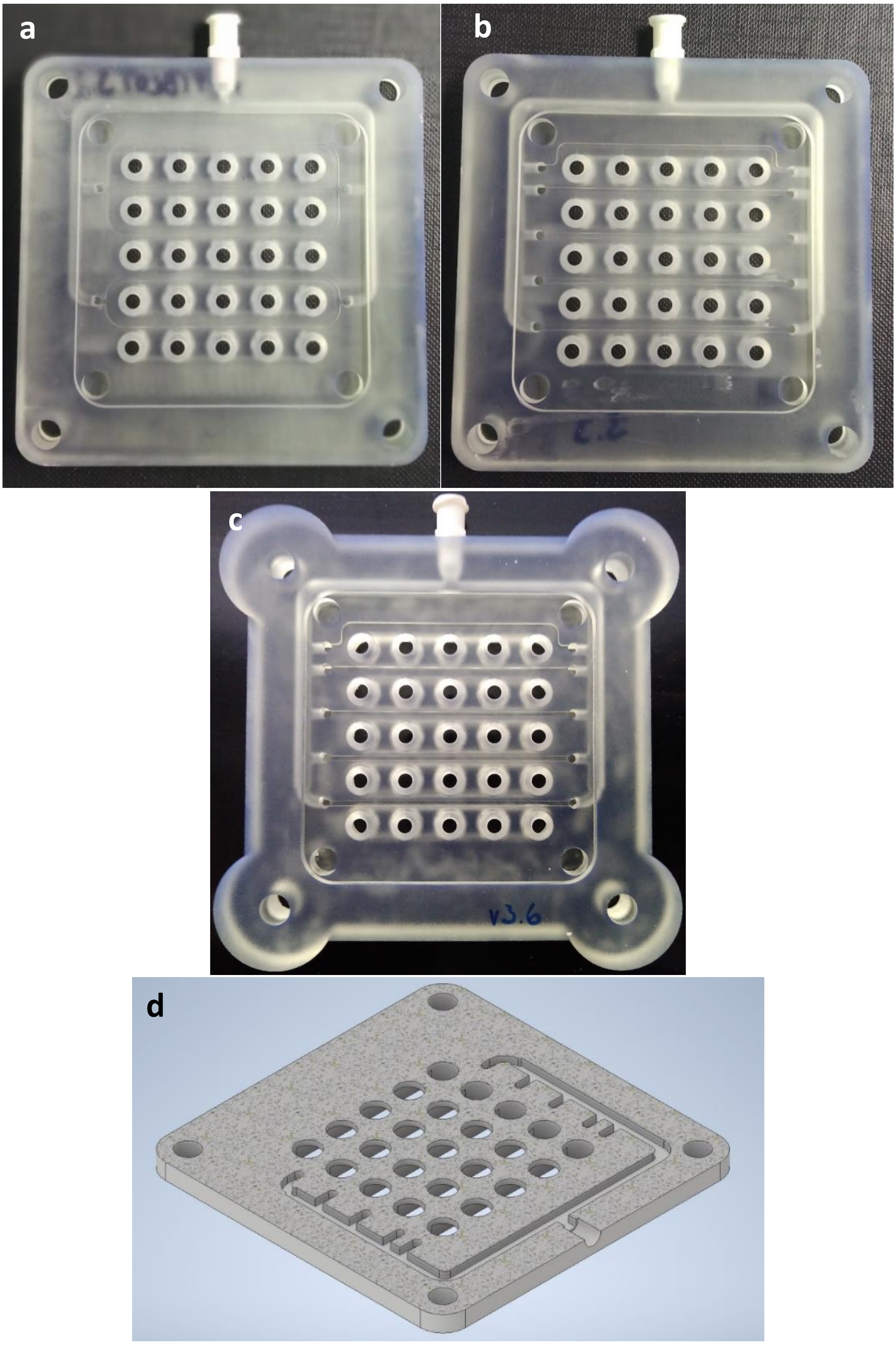

Figure S7. Images of the 4 IO 1 EO (**a**) and the 10 IO 1 EO (**b**) setups, as well as the 10 IO 1EO setup with reinforced screw holes (**c**). The cross-section of the sample manifold (**d**) shows the internal drain system of the 10 IO 1EO setup.

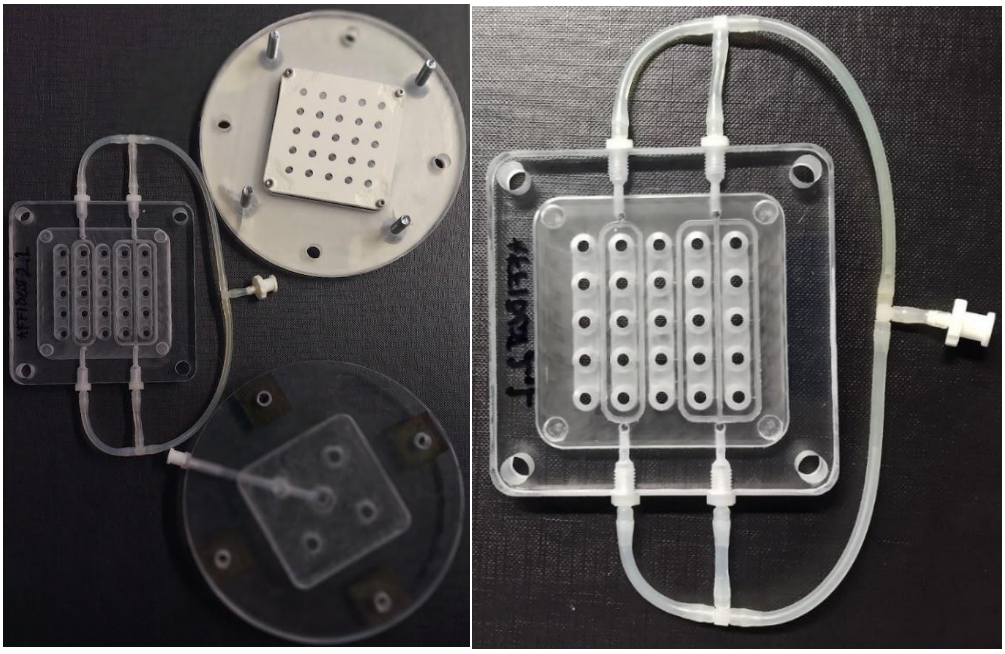

Figure S8. The older, CNC-milled version.

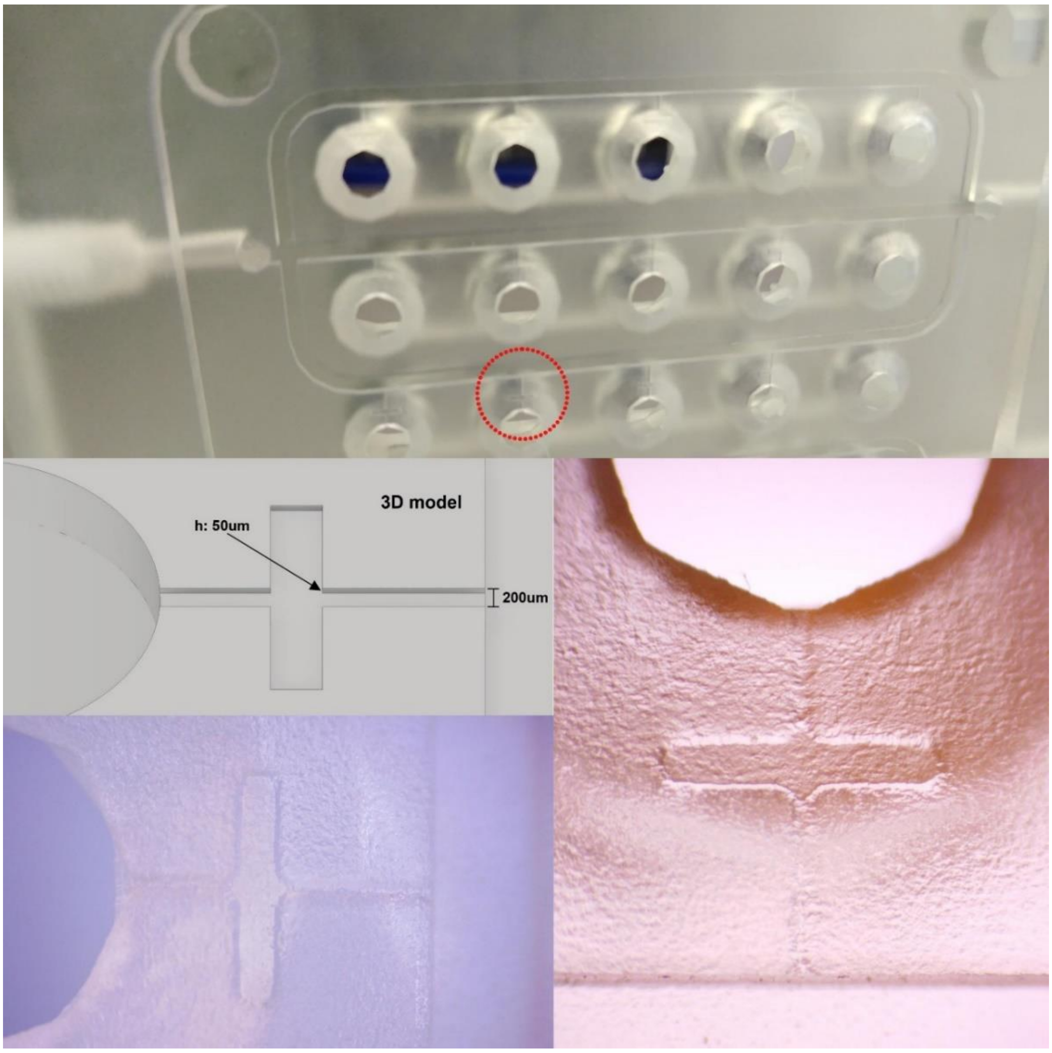

Figure S9. Detail of the 3-and-2 collector channel design (4 IO 1 EO setup). **Lower-right:** Closed-off 200 µm microchannel.
